# PUBLISHING IN AGEING & SOCIETY: TOP 10 TIPS FROM THE EDITOR-IN-CHIEF

**DOI:** 10.1093/geroni/igad104.0954

**Published:** 2023-12-21

**Authors:** Sandra Torres

**Affiliations:** Uppsala University, Uppsala, Uppsala Lan, Sweden

## Abstract

With 43 volumes to its name (and coming out with 12 issues a year), Ageing & Society is an interdisciplinary and international journal devoted to advancing the understanding of ageing, and the circumstances of older people, in their socio-economic and cultural contexts. Committed to publishing original and high-quality research papers that substantially contribute to ongoing debates in social gerontology, this journal welcomes submissions that use different theoretical and methodological approaches as long as they aim to advance research, policy and practice and encourage the exchange of ideas across the broad audience of multidisciplinary academics and practitioners working in the field of ageing. By bringing specific attention to what Ageing & Society looks for in the hundreds of submissions that are sent to the journal, and the routines that the team of associate editors utilize, this presentation aims to give attendees valuable insights into how to write a paper if they wish to submit it to this journal.

